# HSP90 inhibitor 17AAG attenuates sevoflurane-induced neurotoxicity in rats and human neuroglioma cells via induction of HSP70

**DOI:** 10.1186/s12967-020-02332-w

**Published:** 2020-04-15

**Authors:** Min Liu, Moyun Li, Yu Zhou, Qian Zhou, Yugang Jiang

**Affiliations:** 1 Department of Neurosurgery, The Second Xiangya Hospital of Central South University, No. 139, Changsha City, 410000 Hunan Province People’s Republic of China; 2 Department of General Surgery, The Second Xiangya Hospital of Central South University, Changsha City, 410000 Hunan Province People’s Republic of China

**Keywords:** Anesthetic neurotoxicity, 17-*N*-allylamino-17-demethoxygeldanamycin (17AAG), Heat shock protein, Apoptosis, Oxidative stress, NF-κB

## Abstract

**Background:**

17AAG has been extensively studied for its antitumor effects that protect cells from lethal stress by maintaining protein stability. The role of 17AAG in sevoflurane-induced neuronal injury has never been studied. We aim to investigate the effect of 17AAG on sevoflurane-induced neurotoxicity in vivo and in vitro.

**Methods:**

Sevoflurane-induced hippocampal neuron injury model was established in aged Sprague–Dawley rats. Pretreatment of vehicle or 17AAG was administered prior to sevoflurane inhalation. H4 neuroglioma cells were pretreated with vehicle or 17AAG and exposed to sevoflurane. Apoptosis, oxidative stress, expression of interleukin-6 (IL-6), and activation of the nuclear factor-κB (NF-κB) signaling pathway in H4 cells were examined by Hoechst assay, flow cytometry, Western blot, and immunofluorescent staining. RNA interference against *HSPA1A* was performed to test the function of HSP70 in neuroprotection.

**Results:**

Exogenous 17AAG reduced sevoflurane-induced apoptosis and oxidative stress in rat hippocampal neurons and in H4 cells. In H4 cells, 17AAG suppressed sevoflurane-induced upregulation of IL-6 and activation of NF-κB signaling. 17AAG enhanced sevoflurane-induced upregulation of HSP70 in rat hippocampal neurons and in H4 cells. Conversely, silencing of *HSPA1A* in H4 cells blocked the cytoprotective effect of 17AAG against sevoflurane-induced apoptosis and oxidative stress, and prevented upregulation of IL-6 and activation of NF-κB signaling.

**Conclusions:**

17AAG protects against sevoflurane-induced neurotoxicity in vivo and in vitro via HSP70-dependent inhibition of apoptosis, oxidative stress, and pro-inflammatory signaling pathway.

## Background

Volatile inhalation anesthetics are the most commonly used agents to induce and maintain general anesthesia. While providing sedation necessary for life-saving surgical procedures, inhalation anesthetics has been shown in the last twenty years to produce neurotoxic reactions collectively known as anesthetic neurotoxicity [9, 25], commonly manifested as postoperative cognitive decline and neurodegeneration disorders (such as Alzheimer’s disease and Parkinson’s disease) in adults, and neurodevelopmental impairment and behavioral dysfunctions in pediatric patients [16, 43]. Observational studies in animals and humans have documented neurotoxic changes directly after exposure to commonly used inhalation anesthetics, including widespread apoptosis in rodent and primate brain [3, 12, 15, 17, 31]. Sevoflurane is currently the most commonly used inhalation anesthetic agent in developed nations. Although favored for its rapid onset and minimal airway disturbance, sevoflurane has been implicated in emergence delirium [8] and other neurotoxic effects such as anesthesia-induced developmental neurotoxicity (AIDN) [21, 35]. In aged animals, inhaled sevoflurane impaired spatial learning and memory, partially by injuring the hippocampal neurons [38]. In cultured cells, exposure to sevoflurane increased the expression of pro-inflammatory cytokine interleukin (IL)-6 and activation of nuclear factor-κB (NF-κB) pathway [42]. Given that there is no cure or specific treatment for anesthetic neurotoxicity, identifying novel strategy to prevent and treat sevoflurane-induced neuronal injury is therefore a public health need.

17-*N*-allylamino-17-demethoxygeldanamycin (17AAG) is an analog of the antibiotic geldanamycin. 17AAG has been extensively studied for its antitumor effects as a potent inhibitor of heat shock protein (HSP) 90 (HSP90), a member of the HSP molecular chaperones that protect cells from lethal stress by maintaining protein stability. More recently, it was shown that 17AAG, besides inhibiting HSP90, could induce other redundant molecular chaperones, such as the pro-survival HSP70 and HSP40 [27, 36]. Notably, 17AAG has been found to have neuroprotective effect [20], opening up possibility for treating neurodegenerative diseases. However, the role of 17AAG in sevoflurane-induced neuronal injury has never been studied.

## Methods

### Animal protocols

All animal protocols were reviewed and approved by the Animal Ethics Committee of The Second Xiangya Hospital of Central South University. Animals were obtained from the The Second Xiangya Hospital of Central South University. Hippocampal neuronal injury was induced in aged rats by inhalation of sevoflurane (Jiangsu Hengrui Medicine, China) as previously described [32]. Briefly, 20-month-old, male Sprague–Dawley rats (10 per group) received daily intraperitoneal injection of 17AAG (MCE, USA; 30 mg/kg body weight) or vehicle for 2 days. The methods and concentration of 17AAG were adopted from previous reports [22, 28]. After the second injection, rats were exposed to air with or without 2% sevoflurane via an inhalation chamber for 5 h. The methods and concentration of sevoflurane were adopted from previous reports [4, 10]. All rats were sacrificed 24 h after the second injection. The brains were removed. The hippocampal tissue was excised from the mouse brains for protein extraction and lipid peroxidation assay, and the remaining brain was fixed in 4% paraformaldehyde for histology.

We selected the concentrations of sevoflurane used in relation to lowest concentration for neurotoxic effect in vitro according to previously published research [4, 10, 38]. We selected the concentrations of 17AAG in relationship to its lowest concentration for neuroprotective effects in vitro according to previously published research [22, 28].

### Terminal deoxynucleotidyl transferase dUTP nick end labeling (TUNEL) assay

A hippocampal neuronal injury model has been established previously in aged rats by sevoflurane inhalation [32]. Using hippocampal neuronal injury model, we first explored the role of 17AAG in neuronal injury. Apoptosis in rat hippocampus was evaluated by the TUNEL assay using the In Situ Cell Death Detection kit (Roche Diagnostics, Switzerland). Briefly, fixed rat brains were paraffin-embedded and cut in the coronal plane into 5 *µ*m-thick sections. The sections were dewaxed, permeabilized with 0.1% Triton X-100, and stained with the In Situ Cell Death Detection kit following the manufacturer’s instructions. The nuclei were stained by hematoxylin. Stained hippocampal regions were imaged under a microscope. An apoptotic index for each rat was calculated by averaging the percentage of TUNEL-positive cells (number of TUNEL-positive cells × 100/total number of cells in the same microscopic field) in five random fields (×400 magnification) from each hippocampus.

### Lipid peroxidation assay

Oxidative stress in rat hippocampal tissue was measured by quantification of malondialdehyde (MDA), a natural end product of lipid peroxidation. The hippocampal tissue excised from rat brain was homogenized and centrifuged at 10,000×*g* for 10 min at 4 °C for the collection of the supernatant. After normalizing protein concentration with a BCA Protein Assay Kit (Beyotime Institute of Biotechnology, China), the concentration of MDA in the supernatant was measured using a Thiobarbituric Acid Reactive Substances Assay Kit (Cell Biolabs, USA) following the manufacturer’s instructions.

### Cell culture

H4 human neuroglioma cell line was obtained from the Cell Bank of the Chinese Academy of Sciences, China. H4 cells were cultured in RPMI-1640 medium (Gibco Life Technologies, USA) supplemented with 10% fetal bovine serum (HyClone, USA) in a humidified incubator kept at 37 °C with 5% CO_2_, and replaced with fresh medium every 2–3 days. To explored the role of 17AAG in neuroprotection in vitro, we pre-treated H4 cells, a human neuroglia cell line, with either vehicle or 17AAG before exposure to air with or without 4.1% sevoflurane.

### Cell transfection and treatments

Small interfering RNA (siRNA) specifically targeting *HSPA1A* (5′-CCAUGACGAAAGACAACAA-3′) or non-targeting, control siRNA (5′-CGCGUAAGGUCGAAUGCAUAA-3′) were transfected into H4 cells using Lipofectamine RNAiMAX reagent (Invitrogen, USA) following the manufacturer’s protocol. Forty-eight hours after transfection, H4 cells received treatment in the following groups: (1) control group receiving vehicle; (2) 17AAG group; (3) sevoflurane group; and (4) 17AAG+ sevoflurane group. 17AAG (MedChemExpress, USA) was dissolved in DMSO and added to the culture medium as pretreatment for 1 h (final concentration 500 nM) prior to sevoflurane exposure. Cell in control and sevoflurane groups were pretreated with equal volume of DMSO. Pretreated cells were exposed to air with or without 4.1% sevoflurane inside an incubation chamber for 6 h, as described previously [32].

### Apoptosis assay

The apoptosis of H4 cells were determined using a Hoechst staining kit (Beyotime Institute of Biotechnology, China). Briefly, cells were plated on 12-well plates and received transfection and treatments. The cells were fixed in 4% paraformaldehyde, washed with phosphate-buffered saline (PBS), and stained with the Hoechst staining kit following the manufacturer’s protocol. After extensive washing, Hoechst staining was imaged under a fluorescence microscopy (×400 magnification; Zeiss LSM 780, Germany).

### Flow cytometry

The apoptosis of H4 cells was further determined by flow cytometry using an Annexin V-FITC Apoptosis Detection Kit (Thermo Fisher Scientific, USA). Briefly, H4 cells were detached, washed, and gently resuspended in 500 µL binding buffer. Annexin V-FITC (5 µL) and propidium iodide (PI; 5 µL; counterstain) were added to the cell suspension. The mixture was incubated for 10 min at room temperature, washed, and resuspend in binding buffer. Flow cytometry was immediately performed on a flow cytometer (FACSCalibur; BD Biosciences, USA).

### Reverse transcription-quantitative polymerase chain reaction (RT-qPCR)

Total RNA was extracted using the TRI Reagent (MRC, UK). cDNA was synthesized using the SuperScript III First-Strand Synthesis System (Thermo Fisher Scientific). The reverse transcription condition was as follows: 25 °C 10 min, 50 °C 50 min, and 85 °C 5 min. The qPCR reaction was performed in triplicate using the Maxima SYBR Green qPCR Master Mix (Fermentas GmbH, Germany) on a LightCycler 480 Real-Time PCR System (Roche, Germany), with 10 ng cDNA input in 20 uL reaction volume.

Specific primers for human Hsp70 was: (sense: 5-AGGCCAACAAGATCACCATC, antisense: 5-TCGTCCTCCGCTTTGTACTT) and hβ-actin (sense: 5-TGGCACCCAGCACAATGAAG, antisense: 5-GACTCGTCATACTCCTGCTTGC), and mouse Hsp70 rHsp70 (sense: 5-ATGCGCTCGAGTCCTACGCCTT, antisense: 5-GCTGATCTTGCCCTTGAGACCCTC) and rβ-actin (sense: 5-GGAGA TTACTGCCCTGGCTCCTAGC, antisense: 5-GGCCGG ACTCATCGTACTCCTGCTT). The condition for qPCR was 95 °C 10 min, followed by 50 × 3-step cycles: 95 °C 15 s, 59 °C 30 s, and 72 °C 30 s. The relative expression of mRNA was calculated with the ΔΔCq method [6] using hβ-actin or rβ-actin as reference gene for human and rat mRNA, respectively.

### Western blot

Total protein was extracted from hippocampal tissue using RIPA lysis buffer (Beyotime Institute of Biotechnology, China), and from cultured H4 cells using NP-40 lysis buffer (Beyotime Institute of Biotechnology, China). Nuclear and cytosolic proteins were separated using the Nuclear and Cytoplasmic Protein Extraction Kit (Beyotime Institute of Biotechnology, China), and normalized with a BCA Protein Assay Kit (Beyotime Institute of Biotechnology, China). Forty µg protein from each sample was mixed with Laemmli sample buffer, boiled, and resolved by sodium dodecyl sulphate–polyacrylamide gel electrophoresis (SDS-PAGE). Resolved protein was transferred onto a polyvinylidene difluoride membrane (Millipore, USA) and blocked with 5% non-fat milk at room temperature for 1 h. The membrane was incubated with the following primary antibodies: anti-Hsp70 (1:1000; ab2787, abcam, UK), anti-p-IκBα (1:500; bs-5515R, BIOSS, China), and the following all at 1:1000 dilution (Santa Cruz Biotechnology, USA): anti-caspase-3 (sc-56053), anti-Bax (sc-7480), anti-Bcl-2 (sc-7382), anti-IL-6 (sc-130326) and anti-NF-κB (sc-8008) overnight at 4 °C. The membrane was washed and incubated with horseradish peroxidase-conjugated secondary antibodies (A0208 and A0216; Beyotime Institute of Biotechnology, China) for 45 min. Immunoblots were captured on X-ray films by enhanced chemiluminescence reagent (7SeaPharmTech, China) and analyzed by the Gel-Pro Analyzer software for band intensity. The antibodies used are equally reactive against both species (humans and rats).

The membrane was stripped and re-blotted with anti-IκBα (sc-1643, Santa Cruz Biotechnology, USA), anti-β-actin (sc-69879; Santa Cruz Biotechnology, USA), or anti-histone H3 (bsm-33042 M; BIOSS, China) overnight at 4 °C, followed by incubation with secondary antibodies and exposure on X-ray films as above.

### Reactive oxygen species (ROS) assay

Intracellular level of ROS in cultured H4 cells was measured with an ROS Assay Kit (Beyotime Institute of Biotechnology, China). Briefly, a cell-permeable, non-fluorescent probe 2′,7′-dichlorodihydrofluorescein diacetate (DCFH-DA) was added to cell culture in serum-free medium at a final concentration of 10 *µ*M. After incubation for 20 min at 37 °C, extracellular DCFH-DA was washed off, and DCFH, the fluorescent hydrolysis product of DCFH-DA, were trapped intracellularly and was analyzed by flow cytometry as described above.

### Immunofluorescence staining

Cells were fixed in 4% paraformaldehyde for 15 min at room temperature, rinsed with PBS for 3 times, and permeabilized with 0.1% Triton X-100 in PBS. Cells were blocked with goat serum and incubated with anti-NF-κB p65 antibody (1:100; sc-8008, Santa Cruz Biotechnology, USA) overnight at 4 °C. Cells were rinsed, and incubated with a goat-anti-rabbit FITC-conjugated antibody (1:200; A0562, Beyotime Institute of Biotechnology, China) for 1 h at room temperature, and 4′,6-diamidino-2-phenylindole (DAPI) to stain nuclei. Cells were imaged using a fluorescence microscopy (×400 magnification; Zeiss LSM 780, Germany).

### Statistics

Data are expressed as mean ± standard deviation (SD). Difference of means among groups were compared by one-way analysis of variance (ANOVA), followed by Bonferroni multiple comparisons test. A multiplicity adjusted P < 0.05 was considered statistically significant. All data was processed using GraphPad Prism 5 software.

## Results

### 17AAG reduced sevoflurane-induced apoptosis and oxidative stress in rat hippocampal neurons

**Fig. 1:**
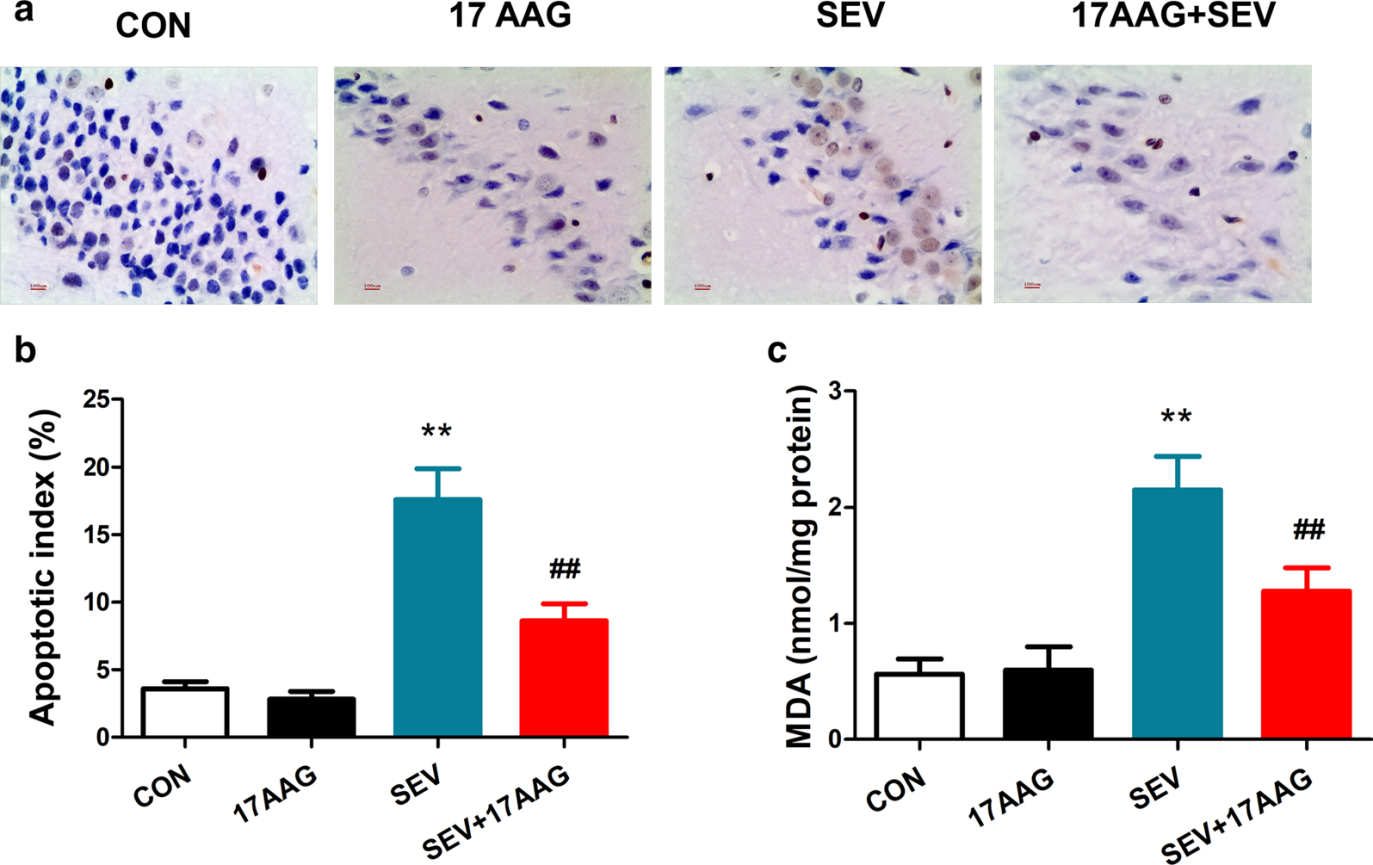
17AAG reduced sevoflurane-induced apoptosis and oxidative stress in rat hippocampal neurons. Male, 20-month-old Sprague–Dawley rats were exposed to 2% sevoflurane (SEV) inhalation with or without receiving a prior 2-day intraperitoneal injection of 17AAG. The hippocampus tissue was removed after the inhalation and sectioned or homogenized. **a** The apoptosis of hippocampal neurons were evaluated on coronal sections with the TUNEL assay and hematoxylin stain (blue, nuclei; brown, TUNEL-stain; ×400 magnification; scale bar, 100 μm). **b** The apoptotic index was calculated by averaging the percentage of TUNEL-positive cells (number of TUNEL-positive cells × 100/total number of cells in the same microscopic field) in five random fields (×400 magnification). **c** The oxidative stress in hippocampal neurons was measured with the MDA assay using normalized tissue lysate. CON, control. n = 10. Mean ± SD, **P < 0.01 vs. CON, ## P < 0.01 vs. SEV by one-way ANOVA followed by Bonferroni multiple comparisons test. The images from Fig. 1a were taken from the same region of dentate gyrus of the hippocampus. The dentate gyrus contains densely packed granule cells

Using hippocampal neuronal injury model, we first explored the role of 17AAG in neuronal injury. The images from Fig. 1a were taken from the same region of dentate gyrus of the hippocampus. The dentate gyrus contains densely packed granule cells. Inhalation of 2% sevoflurane caused acute loss of hippocampal neurons (Fig. 1a) and significant increase of apoptotic cells (Fig. 1a, b) as indicated by hematoxylin and TUNEL staining, respectively. However, rats receiving 2-day intraperitoneal injection of 17AAG showed less neuronal loss and less TUNEL-positive cells after sevoflurane inhalation (Fig. 1a, b). Similarly, 17AAG attenuated the oxidative stress following sevoflurane-injury in hippocampal tissue, as measured by the level lipid peroxidation product MDA in hippocampal tissue (Fig. 1c).

### 17AAG attenuated sevoflurane-induced apoptosis and oxidative stress in cultured H4 cells

**Fig. 2:**
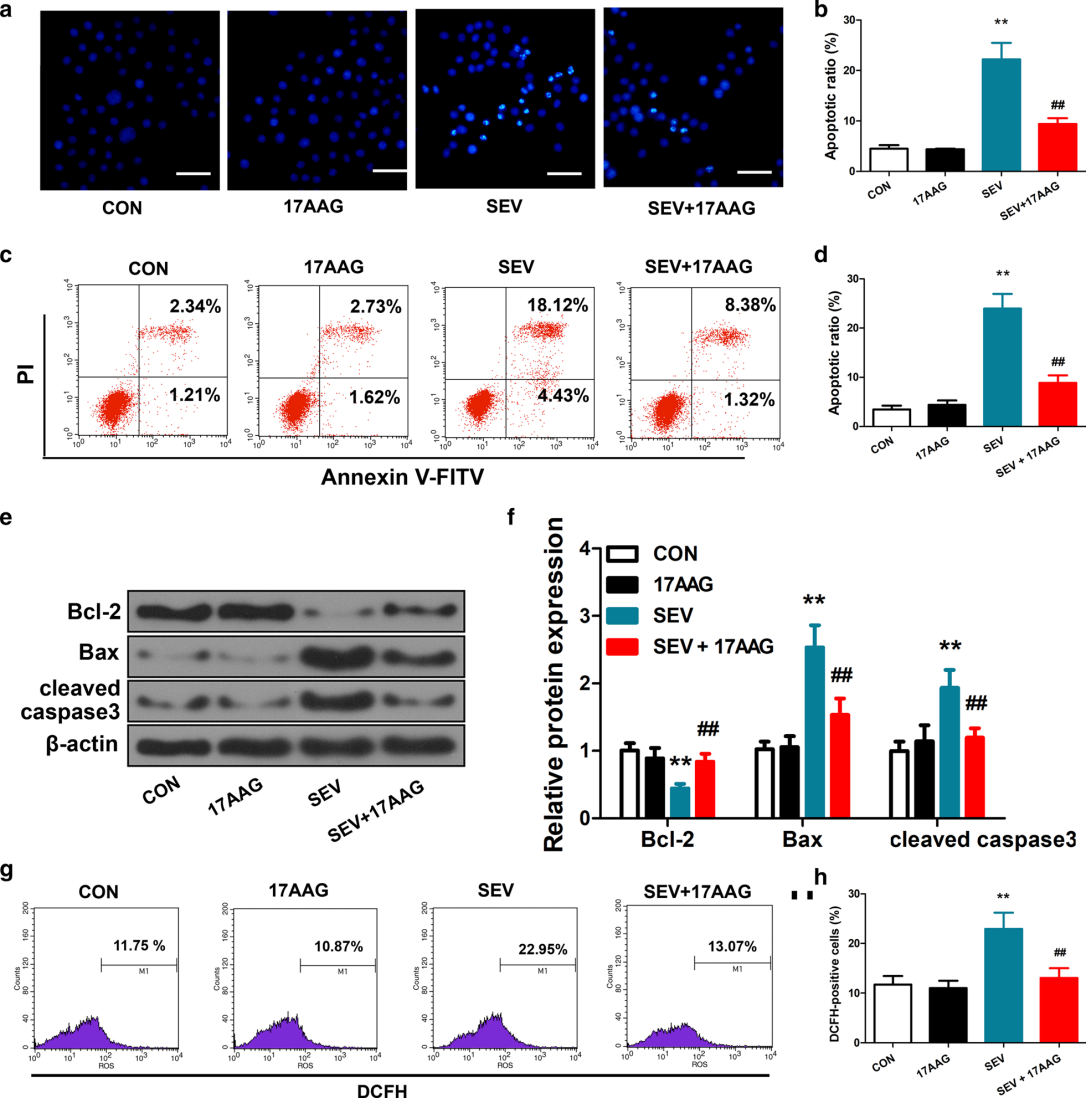
17AAG attenuated sevoflurane-induced apoptosis and oxidative stress in cultured H4 cells. H4 cells were treated with either vehicle (CON) or 17AAG before exposure to air with or without 4.1% sevoflurane (SEV) for 6 h. Cells were analyzed by (**a**, **b**) Hoechst assay (× 400 magnification; scale bar, 50 μm), and (**c**, **d**) flow cytometry to evaluate apoptosis (lower right quadrant, early apoptosis; upper right quadrant, late apoptosis). **e**, **f** The expression of Bcl-2, Bax and cleaved caspase-3 protein in H4 cells was examined by Western blot, with β-actin as loading control. **g**, **h** The generation of reactive oxygen species (ROS) in H4 cells was labeled by a cell-permeable ROS-probe DCFH-DA. The fluorescent hydrolysis product, DCFH, was quantified by flow cytometry. CON, control. Representative of 3 independent experiments. n = 6. Mean ± SD, **P < 0.01 vs. CON, ## P < 0.01 vs. SEV by one-way ANOVA followed by Bonferroni multiple comparisons test

Direct exposure of H4 cells to sevoflurane for 6 h drastically increased cell apoptosis, as measured by Hoechst staining (Fig. 2a, b) and flow cytometry following Annexin V-FITC and PI labeling (Fig. 2c, d). Both early and late apoptosis was increased by sevoflurane exposure (Fig. 2c; lower right quadrant, early apoptosis; upper right quadrant, late apoptosis). Pretreatment of H4 cells with 17AAG had no effect on apoptosis at baseline, but significantly reduced apoptotic cells after sevoflurane treatment (Fig. 2a–d). In line with the effect on apoptosis, sevoflurane marked induced expression of pro-apoptotic protein Bax and the executioner protein of apoptosis, cleaved caspase-3, and downregulated the anti-apoptotic protein Bcl-2. Pretreatment with 17AAG reversed these effects on apoptotic protein expression (Fig. 2e, f).

We also determined the oxidative stress in H4 cells after treatments. Sevoflurane exposure increased generation of ROS, as measured by the ROS-probe DCFH-DA. Pretreatment with 17AAG significantly reduced the level of sevoflurane-induced intracellular ROS (Fig. 2g, h).

### 17AAG inhibited sevoflurane-induced neuroinflammation

**Fig. 3:**
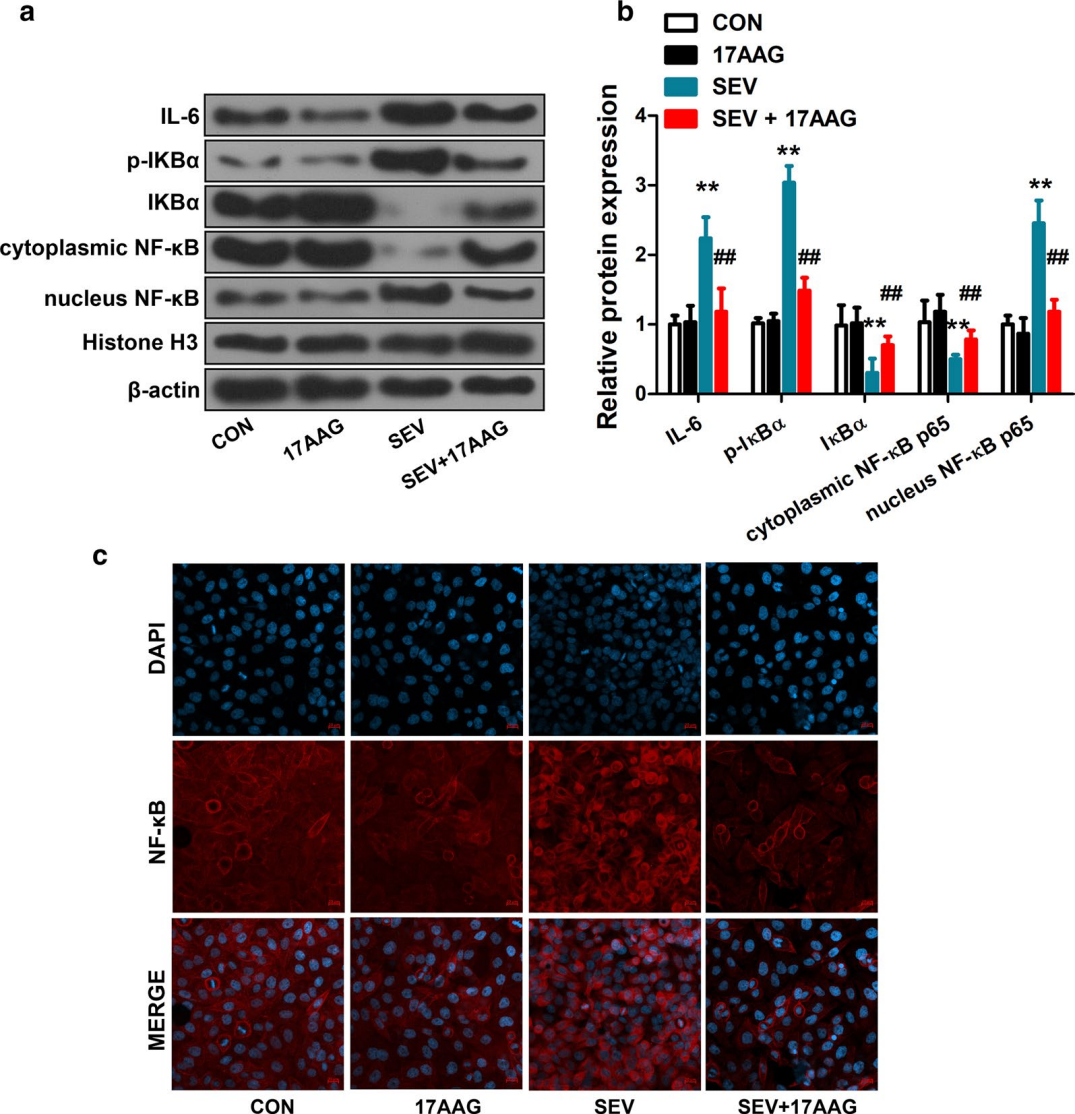
17AAG inhibited sevoflurane-induced upregulation of IL-6 and activation of NF-κB signaling pathway. H4 cells were treated as in Fig. 2. **a**, **b** The expression of IL-6 and proteins of the NF-κB signaling pathway (p-IκBα, IκBα, cytosolic NF-κB p65 and nucleus NF-κB p65) were examined by Western blot using whole or fractionated H4 cell lysate. Histone H3 and β-actin were used as loading controls for nucleus and cytoplasmic extracts, respectively. **c** Immunofluorescence staining of NF-κB p65 distribution in treated H4 cells (×400 magnification; scale bar, 20 μm). Representative images from 3 independent experiments. n = 6 (**a**, **b**). Mean ± SD, **P < 0.01 vs. CON, ## P < 0.01 vs. SEV by one-way ANOVA followed by Bonferroni multiple comparisons test

We exposed H4 cells to 4.1% sevoflurane, a dose that had been previously shown to induce inflammation in neuroglioma cells [42]. We observed significant upregulation of IL-6 and activation of NF-κB pathway, as indicated by increased expression of phospho-IκBα (p-IκBα), decreased expression of IκBα, and translocation of cytosolic NF-κB p65 to nucleus (Fig. 3). Pre-incubation of H4 cells in 17AAG, however, effectively blocked these sevoflurane-induced neuroinflammatory responses, as detected by Western blot and imaging (Fig. 3).

### 17AAG enhanced upregulation of HSP70 in response to sevoflurane injury in vivo and in vitro

**Fig. 4:**
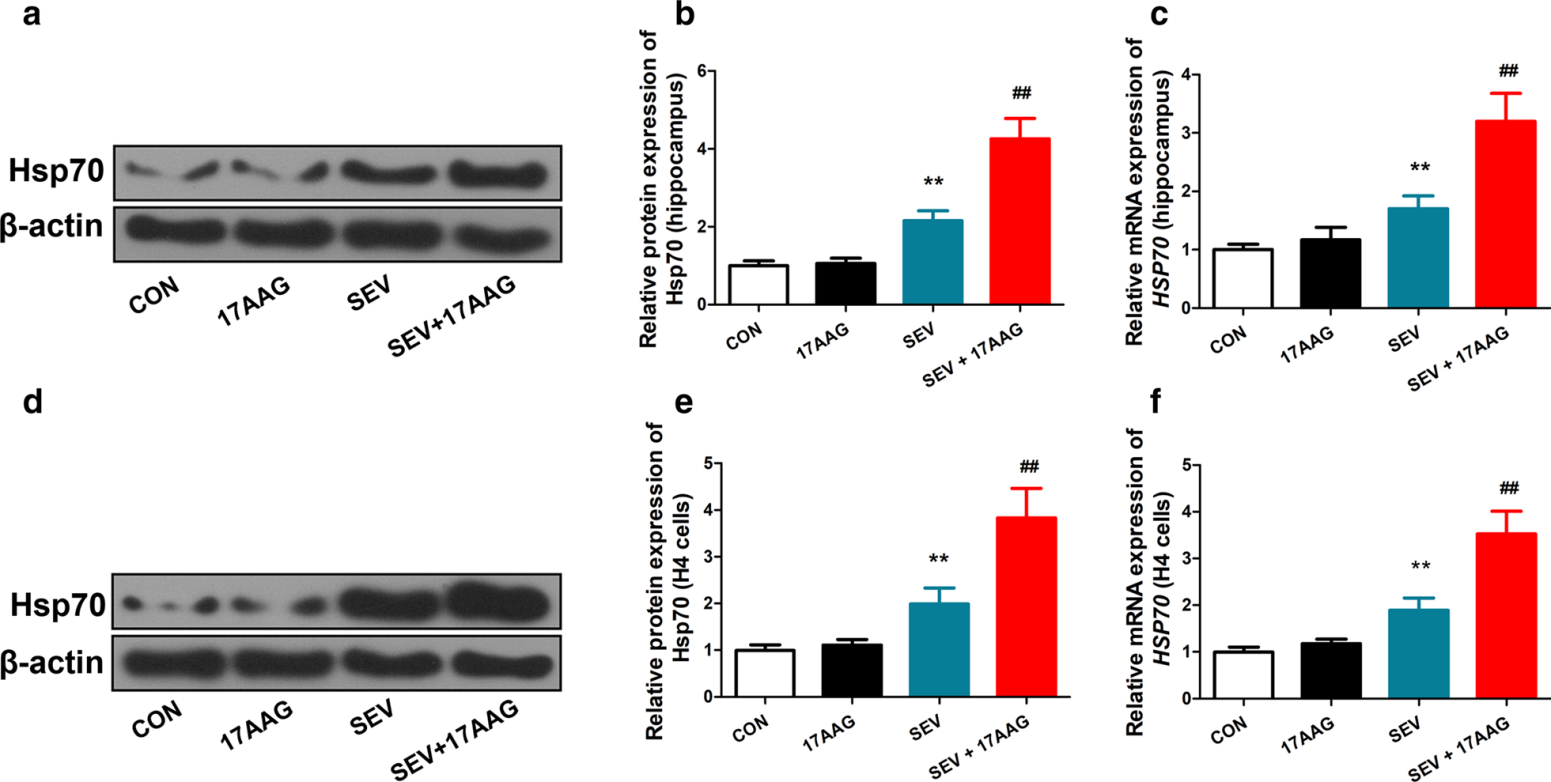
17AAG enhanced upregulation of HSP70 in response to sevoflurane injury in vivo and in vitro. Total protein was extracted from rat hippocampal tissue or cultured H4 cells that were treated as in Figs. 1 and 2, respectively. The expression of HSP70 protein was analyzed by Western blot (**a** and **b**, rat hippocampus; d and e, H4 cells), with β-actin as loading control. The expression of **c** human *HSPA1A* (human) and **f** rat *Hspa1a* mRNA was measured by RT-qPCR. n = 3. Mean ± SD, **P < 0.01 vs. CON, ## P < 0.01 vs. SEV by one-way ANOVA followed by Bonferroni multiple comparisons test

In both rat hippocampal tissue and cultured H4 cells, exposure to sevoflurane led to upregulation of HSP70 (Fig. 4). Pretreatment with 17AAG further enhanced the HSP70 induction by nearly two folds (Fig. 4). These results suggest that upregulation of HSP70 may play a critical role in the neuroprotection by 17AAG. 17AAG further upregulated the expression of HSP70, which was initially induced by sevoflurane.

### HSP70 was required for the cytoprotective effect of 17AAG against sevoflurane-induced neuronal injury

**Fig. 5:**
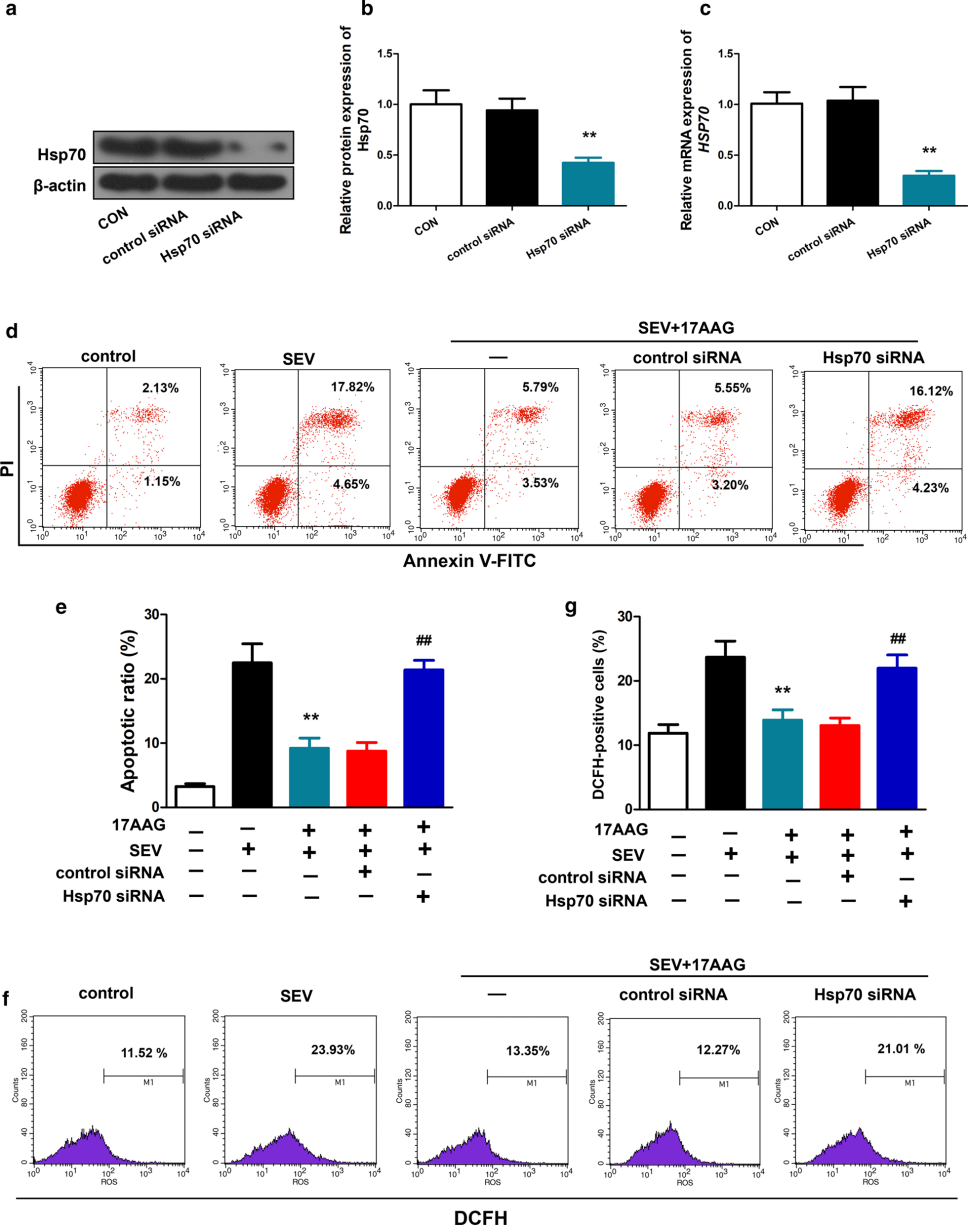
Silencing of HSP70 abolished the cytoprotective effect of 17AAG against sevoflurane-induced apoptosis and ROS generation in H4 cells. H4 cells were transfected with vehicle (CON, control), or siRNA against human *HSPA1A* (*HSPA1A* siRNA), or non-targeting siRNA (control siRNA), and received treatments as in Fig. 2**a**–**c** The expression of HSP70 protein (**a**, **b**) and *HSPA1A* mRNA **c** in H4 cells was examined by Western blot and RT-qPCR, respectively. **d**, **e** Treated cells were stained with Annexin V-FITC and propidium iodide (PI) and analyzed for apoptosis by flow cytometry (lower right quadrant, early apoptosis; upper right quadrant, late apoptosis). (f and g) The generation of ROS in H4 cells was analyzed by flow cytometry as in Fig. 2. Shown representative images of 3 independent experiments (**a**, **d**, **g**). n = 6. Mean ± SD, **P < 0.01 vs. SEV, ## P < 0.01 vs. SEV + 17AAG by one-way ANOVA followed by Bonferroni multiple comparisons test

Given the involvement of HSP70 in neuroprotection against sevoflurane injury in vivo and in vitro (Fig. 4), we further investigated the role of HSP70. siRNA designed against *HSPA1A* could specifically knock-down HSP70 expression in H4 cells (Fig. 5a–c). As observed previously, 17AAG attenuated sevoflurane-induced apoptosis (Fig. 5d, e) and ROS generation (Fig. 5f, g) in H4 cells. However, silencing of *HSPA1A* almost completely abolished these cytoprotective effect of 17AAG (Fig. 5d–g). Importantly, the non-targeting control siRNA had no effect on neuroprotection by 17AAG (Fig. 5).

**Fig. 6:**
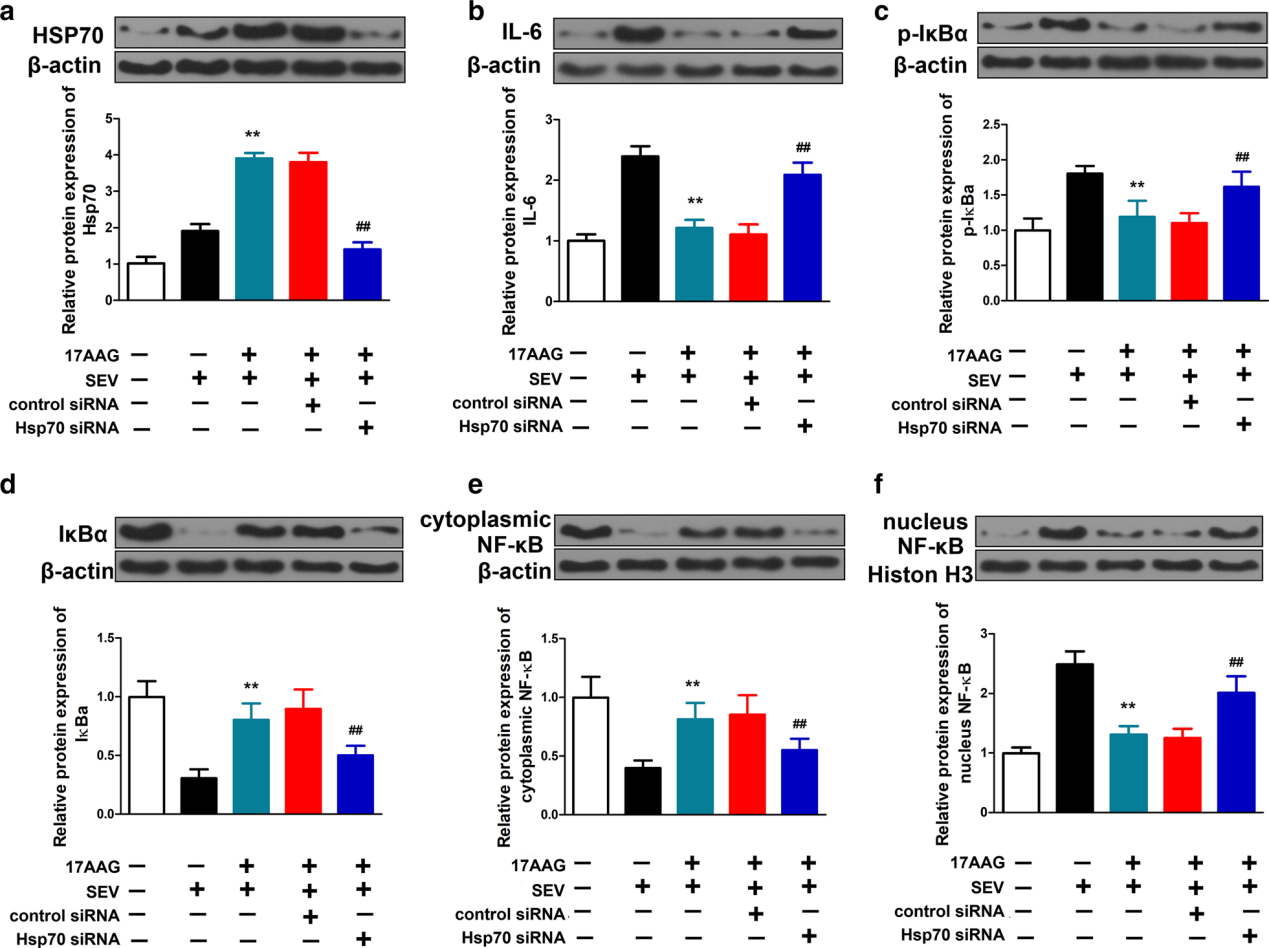
Silencing of HSP70 blocked the inhibition of 17AAG on sevoflurane-induced upregulation of IL-6 and activation of NF-κB signaling pathway. H4 cells were transfected with vehicle, or siRNA against human *HSPA1A* (*HSPA1A* siRNA), or non-targeting siRNA (control siRNA), and received treatments as in Fig. 2. The expression of (**a**) HSP70, (**b**) IL-6, (**c**) p-IκBα, (**d**) IκBα, (**e**) cytoplasmic NF-κB and (**f**) nucleus NF-κB proteins were analyzed by Western blot. β-actin and Histone H3 were used as loading controls for cytoplasmic and nucleus extracts, respectively. Shown representative blots of 3 independent experiments. n = 6. Mean ± SD, **P < 0.01 vs. SEV, ## P < 0.01 vs. SEV + 17AAG by one-way ANOVA followed by Bonferroni multiple comparisons test

In contrast to Fig. 4, siRNA against *HSPA1A* effectively prevented upregulation of HSP70 in response to sevoflurane exposure in H4 cells (Fig. 6). In the absence of *HSPA1A* siRNA, pretreatment with 17AAG inhibited sevoflurane-induced IL-6 upregulation (Fig. 6b) and NF-κB activation (Fig. 3, Fig. 6c–f). However, prevention of HSP70 induction by siRNA blocked the protective effects of 17AAG. The inhibition of IL-6 expression and NF-κB pathway by 17AAG were abolished by siRNA against *HSPA1A*, but not control siRNA (Fig. 6b–f).

## Discussion

The major finding of the present study is that 17AAG confers potent neuroprotective effect against sevoflurane-induced injury. 17AAG effectively attenuated sevoflurane-induced neuronal apoptosis and oxidative stress in vitro. The neuroprotective effect of 17AAG was partially due to the induction of HSP70. Such results provided first evidence for 17AAG as a potential therapeutic agent for anesthetic neurotoxicity.

In the US, over 60,000 patients receive general anesthesia per day [18]. Although neurotoxicity associated with inhaled anesthetics is in general rare, it is associated with grave neurofunctional impairments, sometimes displayed later in life [29]. There is no definitive treatment for anesthetic neurotoxicity. The concern for neurotoxicity is particularly high for sevoflurane, given the relatively larger number of documented incidences in patients and in animal studies [35].

Our results confirmed that one of the major pathways leading to sevoflurane-induced neurotoxicity is via increased apoptosis and oxidative stress. Notably, this appears to be a common effect associated with many widely used inhaled anesthetics on tissues beyond neurons [1, 26, 33, 37, 39]. Several lines of evidence may account for these effects, including excessive activation of peripheral neutrophils [37], direct alteration of enzymatic activities such as glutathione peroxidase and superoxide dismutase [34], and suppression of extracellular signal-regulated kinase phosphorylation [40]. Our results highlighting the pivotal role of apoptosis and oxidative stress in neurotoxicity may have implication for intervention, as these pathways are potentially salvageable and many can be targeted by existing neuroprotective agents [5, 7, 30].

As terminally differentiated cells, neurons are particularly sensitive to oxidative stress causing accumulation of misfolded, aberrant proteins. Dysfunction of HSP chaperones can be lethal to neurons, and was shown to be pathogenic to several neurodegenerative disorders [11]. Our results indicate that HSP70 upregulation is required for the neuroprotective effects of an HSP90 inhibitor, 17AAG. This highlighted the functional redundancy between different HSP molecules, and is consistent with prior studies [2, 24]. Induction of pro-survival HSP70 appears to be an off-target effect of HSP90 inhibition via a potential compensatory mechanism between HSP family members [13, 19, 23]. In cancer, induction of HSP70 and other HSPs by 17AAG accounts for the acquired resistance to chemotherapy by HSP90 inhibition [2]. However, in response to neurotoxic stress, this off-target induction of pro-survival HSP70 by 17AAG appears highly efficacious against apoptosis and inflammatory signal activation, and may be therapeutically beneficial. It is conceivable that future development of direct, selective inducers of HSP70 may be desirable to reduce non-specific impact on protein substrates shared with other HSP members.

Our study has limitations. First, as discussed above, 17AAG may have non-specific effects on HSP family members. Our study focuses on HSP70, which cannot exclude the compensatory effect from other HSP client molecules that may also contribute to the neuroprotective effect of 17AAG. Second, sevoflurane-induced neurotoxicity occurs most commonly in pediatric patients [35]. However, our model of sevoflurane-induced neurotoxicity was established in aged rat, which could capture the characteristics of neurodegenerative diseases, but may not accurately reflect the pathogenesis of sevoflurane-induced developmental neurotoxicity in children [21]. Third, given the altered baseline expression of HSP70 family members in neuroglioma, the effects seen in cultured H4 cells may not fully represent those of the primary neurons.

Many studies have reported that H4 neuroglioma cells are used in sevoflurane induced neurotoxicity studies [14, 41]. In this study, we also used H4 neuroglioma cells to investigate 17AAG on sevoflurane-induced neurotoxicity protection. A better model is to use H4 neuroglioma cell lines from the hippocampus, which will be a better representation for the apoptotic effect of sevoflurane on normal hippocampal cells and the counter effects that 17AAG can present. Considering this limitation, we will use H4 neuroglioma cell lines in our future work.

## Conclusions

Taken together, our study found the HSP90 inhibitor, 17AAG, conferred cytoprotection against sevoflurane-induced neurotoxicity in rat hippocampal neurons and in cultured H4 cells. 17AAG inhibited sevoflurane-induced apoptosis, oxidative stress, and activation of inflammatory signaling in neurons, partially via induction of HSP70. Our study provided evidence supporting HSP70 induction may be effective in the management of sevoflurane-induced neurotoxicity.

## Data Availability

The datasets used and analysed during the current study are available from the corresponding author on reasonable request.

## Abbreviations

17-AAG: 17-*N*-allylamino-17-demethoxygeldanamycin
HSP: Heat shock protein
AIDN: Anesthesia-induced developmental neurotoxicity
ANOVA: Analysis of variance
DAPI: 4′,6-diamidino-2-phenylindole
DCFH-DA: Dichlorodihydrofluorescein diacetate
IL-6: Interleukin-6
MDA: Malondialdehyde
NF-κB: Nuclear factor-κB
p-IκBα: Phospho-IκBα
PBS: Phosphate-buffered saline
PI: Propidium iodide
ROS: Reactive oxygen species
RT-qPCR: Reverse transcription-quantitative polymerase chain reaction
SD: Standard deviation
siRNA: Small interfering RNA
TUNEL: Terminal deoxynucleotidyl transferase dUTP nick end labeling
